# Mapping Human Resources to Guide Ophthalmology Capacity-Building Projects in Honduras: Sub-national Analyses of Physician Distribution and Surgical Practices

**DOI:** 10.5334/aogh.4384

**Published:** 2024-03-11

**Authors:** Rocio Banegas, Luis Rojas, Mariela Castillo, Luis Lagos, Kevin Barber, Britton Ethridge, Sara O’Connor

**Affiliations:** 1 Advanced Center for Eyecare Global, 1721 Westwind Drive, Suite B, Bakersfield, CA 93301, US; 2 Advanced Center for Eyecare Global, US; 3 Hospital San Felipe, Tegucigalpa, HN; 4 Centro de Salud Integral ZOE, Tegucigalpa, HN; 5 Advanced Center for Eyecare Global, Alabama College of Osteopathic Medicine, 445 Health Sciences Blvd, Dothan, AL 36303, US

**Keywords:** cataract surgical rate, capacity-building programs, Honduras, poverty

## Abstract

**Objective:**

To map ophthalmologist locations and surgical practices as they vary sub-nationally within Honduras to maximize the impact of efforts to develop cataract surgical capacity.

**Methods:**

An anonymous survey was sent to all Honduran ophthalmologists with questions on surgical volume, department-level location, type of facility in which they work, surgical methods, and age. Surgical volume, population, and poverty data sourced through the Oxford Poverty Human Development Initiative were mapped at the department level, and cataract surgical rates (CSR; surgeries per million population per year) were calculated and mapped.

**Results:**

Sixty-one of the 102 Honduran ophthalmologists contacted responded. Of those, 85% perform cataract surgery, and 49% work at least part time in a non-profit or governmental facility. Honduras has fewer surgical ophthalmologists per million than the global average, and though national CSR appears to be increasing, it varies significantly between departments. The correlation between CSR and poverty is complex, and outliers provide valuable insights.

**Conclusion:**

Mapping ophthalmological surgical practices as they relate to population and poverty at a sub-national level provides important insights into geographic trends in the need for and access to eye care. Such insights can be used to guide efficient and effective development of cataract surgical capacity.

## Introduction

Cataracts are the leading cause of blindness globally and the second most common cause of moderate to severe visual impairment, affecting an estimated 100 million people [1]. All regions are affected; however, approximately 90% of preventable vision loss due to cataracts is in low- and middle-income countries [1]. Although age-standardized rates of blindness are decreasing globally [2], the absolute number of people suffering from vision loss continues to increase due to increased life expectancy and population growth [2].

Cataracts are treated surgically, and international organizations have drawn attention to the need for improved access to surgical eye care [1,3,4,5]. In 2013, the World Health Assembly created a Global Action Plan to achieve universal eye health, and cataract is one of the priority conditions in this plan. In an effort to meet the need, many governments and development organizations are striving to increase cataract surgical capacity. Unfortunately, the growing burden of cataract blindness continues to outpace the training of new ophthalmologists globally [6,7], and the number of ophthalmologists practicing in high-income and urban areas is increasing at a greater rate than in low-income and rural areas [7].

Creating effective surgical capacity-building programs to address unequal access to surgical eye care requires thoughtful development guided by focused needs assessments. One useful metric for measuring the relative supply of and access to eye care is the cataract surgical rate (CSR), defined as the number of cataract surgeries per year per million people. CSR is one of the three core indicators used by the World Health Assembly Global Action Plan to monitor progress toward universal eye health, and it varies globally from 0 in many locations to between 500 and 2000 in parts of sub-Saharan Africa and Asia to over 10 000 in parts of the economically developed world [8]. CSR is a proxy indicator of access to care, it is significantly correlated to both gross domestic product per capita and gross national income per capita [8], and it is typically calculated at a national level.

A specific region’s actual CSR depends upon factors that vary sub-nationally. Locally driven barriers to care, such as difficult terrain or fear of surgery owing to bad outcomes known to a community, can decrease local demand for surgery. Vision impairment is associated with poverty [9] and thus also varies at a sub-national level. Surgical eye care availability also varies, and the presence of an ophthalmologist in a region does not equate to a known level of service provision. Calculating CSR at a sub-national level, exploring the variation, and then using the resulting information to carefully target development efforts will increase the impact of projects focused on building capacity.

Like many developing countries, Honduras has a large population that suffers from vision impairment and blindness due to cataracts. A 2022 population-based study estimated a blindness prevalence of 4.5% among Hondurans aged 60 years or older [10], and the burden of blindness and visual impairment in Honduras is projected to increase as the population ages over the next 30 years [11]. Despite the clear need for ophthalmologic care, Honduras had the lowest CSR of all 19 Latin American countries at 750 surgeries per million per year in 2008 [12]. By 2016, the CSR had increased to 1181 [13], but this relatively low CSR can be explained by the disproportionately small concentration of surgical ophthalmologists in Honduras (4.5 per million), which is well below the global average of 14.1 per million [7]. Additionally, there is only one ophthalmology residency program in the country that trains three residents per year, a low number relative to the size of the population. The burden of blindness in Honduras, combined with the relative paucity of surgical ophthalmologists, calls for an increase in cataract surgical capacity in the country.

The purpose of this study is to map ophthalmologist distribution and practice trends across Honduras and examine how these trends vary when considering population and poverty levels. This information will aid capacity-building programs—including both the government and international eye care organizations—in determining where services are most needed and in providing insight to newly trained ophthalmologists regarding these needs as they choose a location in which to work. Such anaylsis could also help the government plan the development of health care facilities and potentially serve as a model for other capacity-building programs as they approach needs assessments, project development, and impact analysis.

## Materials and Methods

An anonymous electronic survey and a cover letter explaining the goals of the project were sent to 102 ophthalmologists known to be practicing in Honduras between February and August 2023. All registered members of the national organization of ophthalmologists, the Sociedad Hondureña de Oftalmología, were contacted as well as others known to the authors. Email reminders were sent two months after the initial request, and participants were also recruited at the yearly meeting of the national society in May 2023. Additional data were gathered via personal communication with non-Honduran surgical ophthalmologists known to work in Honduras and from international non-profit organizations known to support surgical ophthalmology work in Honduras. Institutional review board approval was obtained through the Ethical Review Committee at the Hospital General San Felipe, and the tenets of the Declaration of Helsinki were followed.

The anonymous survey asked participants to specify the geographic location where they practice (at the department level), the type of facilities in which they work (private, non-government, or public) and the hours spent weekly in each, the number of cataract surgeries they complete per year, cataract surgical technique, and the number of patients they see in each type of facility per year in a normal year not affected by the COVID-19 pandemic. Data on the age of the ophthalmologists was also collected.

ArcGIS was used to create the maps and obtain sub-national population data on Honduras [14]. Poverty data were sourced through the Oxford Poverty and Human Development Initiative [15] and represents data from a 2019 Multiple Indicator Cluster survey. This index of poverty was generated by combining incidence and severity data on 10 indicators of poverty, including nutrition, school attendance, source of drinking water, and household assets, where higher values indicate greater poverty.

## Results

Sixty-one of the 102 (60%) Honduran ophthalmologists who were contacted participated in the study. Additionally, two individuals and two organizations that travel to Honduras and provide surgical eye care provided responses. The international non-governmental organization SEE International also provided the department-level locations and approximate numbers of cataract surgeries supported by their organization.

Of the 61 Honduran ophthalmologists who replied to the study, 85% (n = 52) reported performing surgery, and 10% (n = 6) reported doing cataract surgery but did not provide data on surgical numbers. Regarding practice setting, 51% (n = 32) work only in a private setting, 3% (n = 2) work only in a non-governmental setting, and 5% (n = 3) work only in a public setting. Many physicians work in multiple settings, with the remaining 41% (n = 25) doing at least some of their work in public or non-governmental institutions. In total, Honduran ophthalmologists reported spending 1317 hours per week in private settings, 322 hours per week in non-governmental settings, and 430 hours per week in public settings.

Honduran ophthalmologists reported an estimated total of 15 106 yearly cataract surgeries. Of those surgeries, 37% (n = 5680) are done at a private facility, 47% (n = 7064) are done at a non-governmental facility, and 16% (n = 2362) are done at a public facility. Of the 45 surgeons who reported on their surgical techniques, 22% (n = 10) report doing only phacoemulsification, 22% (n = 10) report doing only manual small incision cataract surgery (MSCIS), and 56% (n = 25) report doing a combination of phacoemulsification and MSICS.

Based on the total number and surgery locations reported by Honduran ophthalmologists (mapped in Figure 1A) and UN population data from 2022 [16] (mapped in Figure 1B), the CSR for Honduras is 1420 cataract surgeries per million per year. Non-Honduran ophthalmologists perform an additional 1854 surgeries per year in Honduras, and when that number is added to the total, the CSR for the country increases to 1594. Department-level CSR, which was obtained using department-level population data from 2020 and survey data, and department-level poverty index values are shown in Table 1 and mapped in Figure 1C. If a surgeon reported working in multiple departments, their total surgery numbers were divided evenly between those departments.

**Figure 1A:**
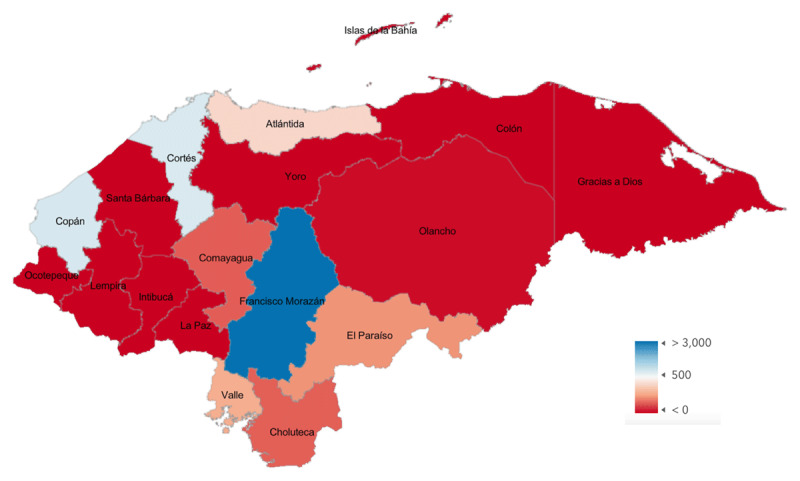
Cataract Surgeries per Year by Honduran Department.

**Figure 1B:**
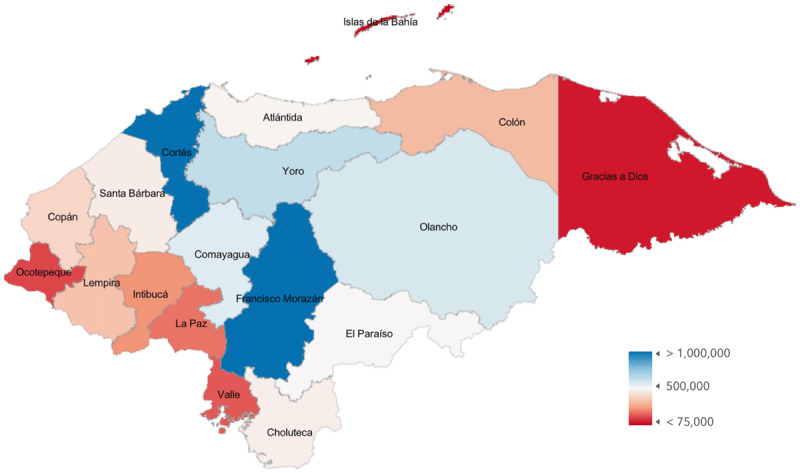
Population by Honduran Department.

**Table 1:** cataract Surgical Rate (surgeries per million per year) and Multidimensional Poverty Index by Honduran Department.


DEPARTMENT	CATARACT SURGICAL RATE (SURGERIES PER MILLION PER YEAR)	MULTIDIMENSIONAL POVERTY INDEX

Islas de la Bahía	0	0.011

Cortés	2906	0.023

Francisco Morazán	4787	0.038

Atlántida	1261	0.057

Colón	0	0.061

Valle	2599	0.091

Yoro	338	0.092

Santa Bárbara	454	0.104

Comayagua	895	0.110

Olancho	26	0.122

Choluteca	303	0.136

Ocotepeque	0	0.136

El Paraíso	460	0.139

La Paz	0	0.154

Intibucá	0	0.189

Copán	3211	0.196

Gracias A Dios	0	0.212

Lempira	0	0.243


**Figure 1C:**
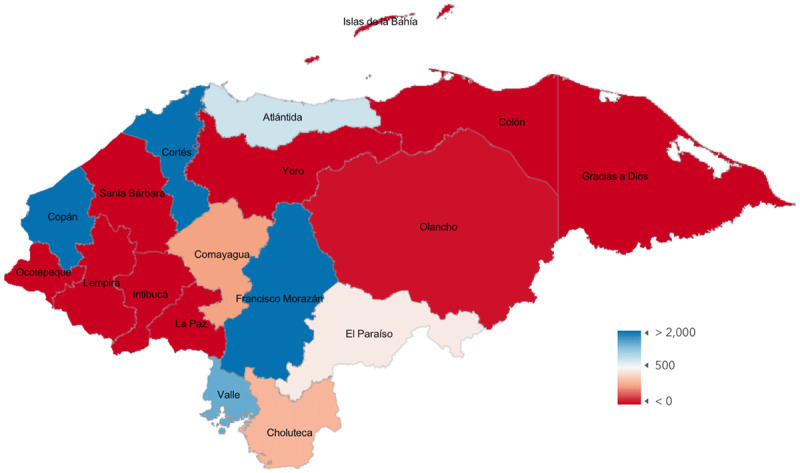
Cataract Surgical Rate (surgeries per million per year) by Honduran Department.

**Figure 2:**
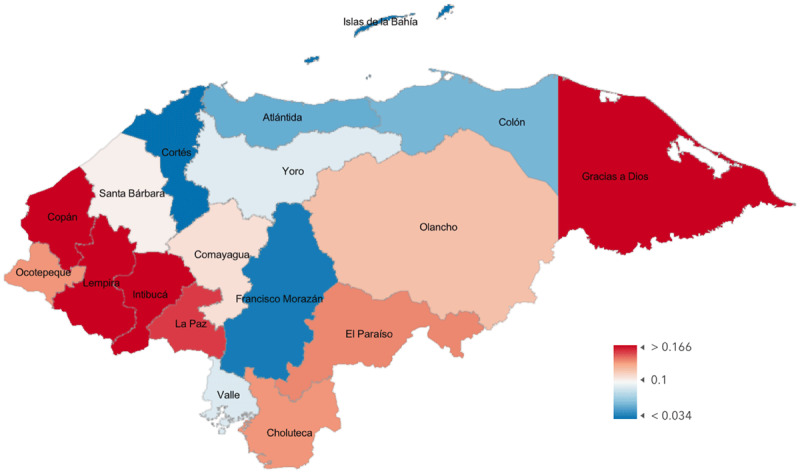
Poverty as Measured by the Multidimensional Poverty Index by Department.

**Figure 3:**
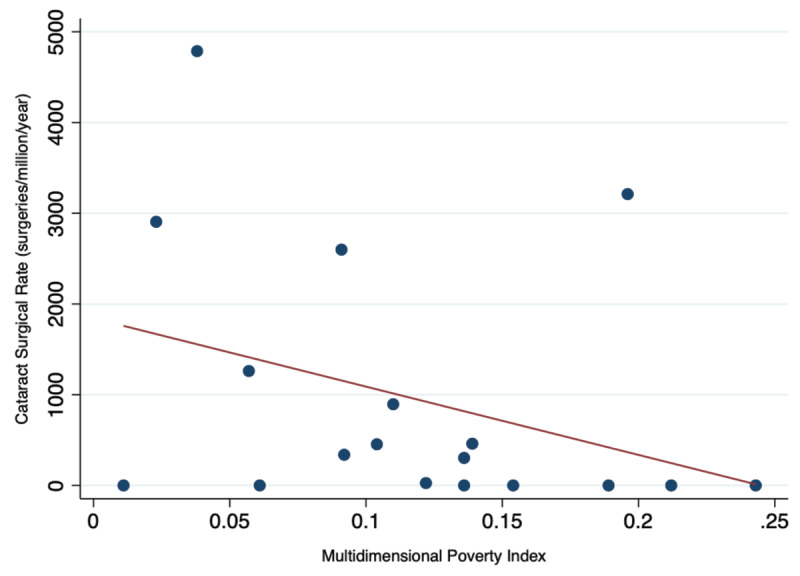
Cataract Surgical Rate vs. Multidimensional Poverty Index in Honduras.

**Figure 4:**
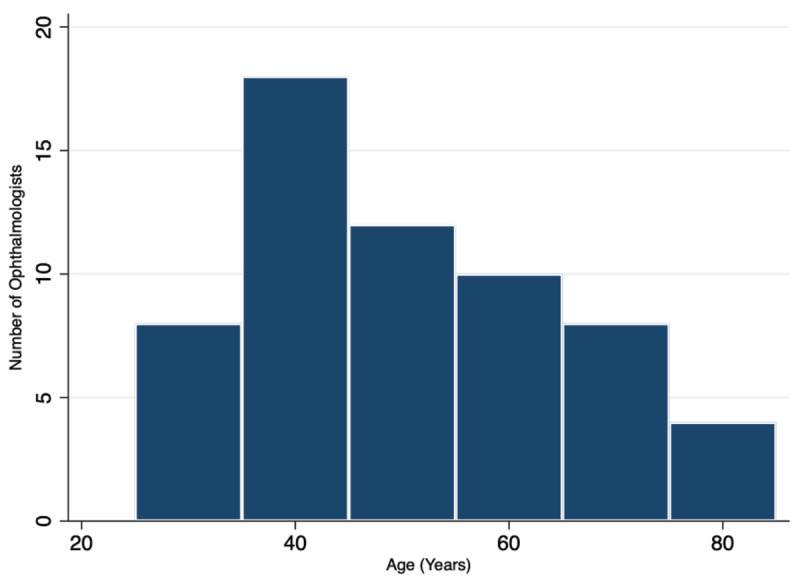
Age of Honduran Ophthalmologists.

Department-level data on poverty in Honduras using the Oxford Multidimensional Poverty Index (MPI) [15] are mapped in Figure 2. The national MPI for Honduras is 0.093, where urban areas have an average MPI of 0.025 and rural areas have an average of 0.155. MPI levels range from 0.011 in Islas de la Bahía to 0.243 in Lempira. The relationship between MPI and CSR by department (Pearson’s *r* = –0.35; *P* = 0.16) is shown in Figure 3. The age distribution of practicing ophthalmologists in Honduras is shown in Figure 4. MPI values from neighboring countries lend these numbers context, ranging from 0.002 in Costa Rica to 0.134 in Guatemala.

## Discussion

This study provides insight into current ophthalmologic surgical practices as they relate to population and poverty in Honduras, and it reveals trends that would be obscured by national-level data.

The total of 102 ophthalmologists translates into 9.6 ophthalmologists per million people in Honduras. If 58 ophthalmologists truly represent the cataract surgical workforce in the country, there are 5.5 surgical ophthalmologists per million people. This is higher than the mean for developing countries of 3.7 per million population [7] and likely represents an underestimate due to survey non-response. However, it is well below the global average of 14.1 per million and has shown only a modest increase since the 2020 estimate of 4.5 surgical ophthalmologists per million in Honduras [7].

In the absence of health care programs for the poor, patients without means rely exclusively on public or non-government facilities for cataract surgery. The estimated 752 hours per week that the surveyed ophthalmologists report working in public or non-governmental facilities is the equivalent of 19 full-time ophthalmologists serving the poor of Honduras. According to the Oxford Poverty and Human Development Initiative [15], 29.1% of Hondurans are vulnerable to, or in, extreme poverty, which translates to approximately 3 million people. If the equivalent of 19 full-time ophthalmologists work in public or non-governmental facilities, there are approximately six ophthalmologists per million people among the poor.

A Honduras CSR of 1594 is higher than previously reported [13]. Additionally, the number of surgeries reported in this study may be artificially low due to non-response by Honduran ophthalmologists and incomplete capture of non-Honduran ophthalmologists working in the country. Even with these limitations, the calculated national CSR appears to be increasing, which is important to recognize and celebrate. A more nuanced story emerges when CSR is calculated at a department level. There are four departments (Cortés, Francisco Morazán, Valle, and Copán) Table 1 in which the CSR is greater than the national CSR, but the remainder fall short of that number, and seven departments have a CSR of 0. Cataract surgical access is, therefore, much more variable than a single national number suggests, and sub-national analysis improves insight into the areas with the greatest need.

Maps depicting department-level CSR allow visualization of the magnitude and distribution of the inequities and can be used to inform program development. Areas with low access adjacent to areas with greater access could benefit from the creation of small satellite facilities. The provision of transportation between such areas could provide access to the underserved and avoid the need to build new facilities. Geographic proximity makes short-term surgical expeditions with trained staff providing follow-up care more logistically feasible. Understanding the sub-national distribution of service provision allows for creative program development that increases access to care for the populations in greatest need.

One way to use CSR is to set an attainable target value, estimate the magnitude of increase needed in a specific area to meet that target, and then tailor program development with that value in mind. Inherent in setting a target CSR is the understanding that the number of surgeries needed each year includes the incident cases of cataract-related visual impairment and the prevalent cases that have been unoperated due to lack of access to care. Therefore, useful target values for CSR vary based on disease prevalence, the visual acuity at which cataracts are treated, and disease incidence as a function of the age structure of the population [17,18]. In a study in which target CSR was modeled in 19 African countries, values varied between 1200 and 4500 [19]. Another study modeling target CSR in Latin America produced figures ranging from 3441 to 8935 [18].

The age structure of the population is one of the primary drivers of a target CSR number, and Lewallen [19] suggests that age structure alone could be used to estimate a target CSR. A recent population analysis indicates that approximately 13% of the Honduran population is over 50 years [20]. In the above studies, the countries most similar to Honduras are Guatemala and Ghana. The Latin American study [18] calculated a target CSR of 4354 for Guatemala, a country in which 12.3% of the population is older than 50 years. The study on Africa [19] calculated a target CSR of 2868 for the eastern region of Ghana, an area in which 11.9% of the population is over 50 years. Using these numbers and the calculated sub-national CSR numbers in the Table to extrapolate an attainable CSR for Honduras, 3000 could be used as an initial, very conservative target CSR for Honduras. As progress is made toward this goal, the target CSR could increase to more ambitious levels, which would fully address the entire burden of preventable blindness.

Comparison of sub-national CSR values with poverty levels provides further insight into geographic trends in CSR, as it indicates that CSR tends to be lower in more impoverished areas. The inverse correlation between poverty and CSR, though not quite statistically significant when considering all data points, suggests that areas of greater poverty have less access to eye care. Outliers, however, distort the relationship and are worth considering. For example, Islas de la Bahia is a relatively affluent area, but per our surveys, no surgery is done there. This could be explained by either our failure to capture survey data or the fact that people of the area are sufficiently affluent to be able to travel to have cataract surgery. The other outlier is Copán, one of the departments with the highest MPI scores and in which the CSR is relatively high. This is largely due to the presence of a single practice, and while it is an excellent example of the potential for service provision in an impoverished area, it is a clear outlier. When those two outliers are removed, the correlation coefficient increases to –0.65 with a *P* value of 0.01.

Critics of sub-national CSR calculation might suggest that a surgeon’s location does not limit the area they serve. Patients can travel to populated centers where cataract surgery is available in order to access care, making department-level CSR an inaccurate representation of access. However, in Honduras, factors like mountainous terrain, difficult roads, and seasonal flooding limit easy movement for much of the population, effectively limiting access to eye care for a large portion of the population. Similar factors are potentially present in many other developing countries, which makes descriptions of surgeon location and behavior important aspects of understanding regional access. Going forward, other approaches to understanding access, such as tracking patient-level information on distance traveled to specific facilities, would further improve the understanding of regional eye care delivery.

Finally, the age structure of the ophthalmologic workforce in Honduras has a distribution that indicates that the supply of ophthalmologists is stable into the future. Enticing young ophthalmologists to stay in the country to practice remains an important goal in working to build surgical capacity in the developing world.

The main strength of this study is that it provides insight into sub-national access to cataract surgical care, allowing for more strategic development of capacity. It also provides a visual representation of multiple sources of data, giving a more complete and nuanced picture of populations, poverty, and eye care services available.

Incomplete survey response is a weakness of this study. Despite multiple and varied attempts to collect data, the authors were unable to capture complete information on surgical practices. Survey non-response may not have been random, as ophthalmologists who operate may have been more likely to respond to a survey largely about surgical practices. This potentially minimized the distortion of results caused by non-response. There are ongoing attempts to collect complete information.

Sub-national survey data, publicly available datasets on poverty and population distribution, and widely used mapping software packages can be combined to provide valuable insights into the geographic and socioeconomic patterns of eye care access. Ideally, such insights are used to guide effective program development and to maximize the impact of resources devoted to addressing preventable blindness. Similar methods could be used for other capacity-building programs as they approach needs assessments, project development, and impact analysis.

## Data Accessibility Statement

The data that support the findings of this study are available on request from the corresponding author O’Connor. The data are not publicly available due to privacy concerns.
